# A Review of Homomorphic Encryption for Privacy-Preserving Biometrics

**DOI:** 10.3390/s23073566

**Published:** 2023-03-29

**Authors:** Wencheng Yang, Song Wang, Hui Cui, Zhaohui Tang, Yan Li

**Affiliations:** 1 School of Mathematics, Physics and Computing, University of Southern Queensland, Toowoomba, QLD 4350, Australia; 2 School of Computing, Engineering and Mathematical Sciences, La Trobe University, Bundoora, VIC 3086, Australia; 3 Faculty of IT, Claytyon Campus, Monash University, Clayton, VIC 3800, Australia

**Keywords:** biometrics, biometric security, privacy, homomorphic encryption, privacy preserving

## Abstract

The advancement of biometric technology has facilitated wide applications of biometrics in law enforcement, border control, healthcare and financial identification and verification. Given the peculiarity of biometric features (e.g., unchangeability, permanence and uniqueness), the security of biometric data is a key area of research. Security and privacy are vital to enacting integrity, reliability and availability in biometric-related applications. Homomorphic encryption (HE) is concerned with data manipulation in the cryptographic domain, thus addressing the security and privacy issues faced by biometrics. This survey provides a comprehensive review of state-of-the-art HE research in the context of biometrics. Detailed analyses and discussions are conducted on various HE approaches to biometric security according to the categories of different biometric traits. Moreover, this review presents the perspective of integrating HE with other emerging technologies (e.g., machine/deep learning and blockchain) for biometric security. Finally, based on the latest development of HE in biometrics, challenges and future research directions are put forward.

## 1. Introduction

Biometrics is the measurement of human physiological and behavioural characteristics with the purpose of recognising and describing individuals [1]. Biometric traits include biological traits (e.g., fingerprint, face and iris) and behavioural traits (e.g., voice, signature and keystroke). Thanks to the desirable attributes of biometric traits [2], such as distinctiveness, invariance and robustness, biometric systems are now extensively used for identity verification in many applications (e.g., e-health, e-banking and border control). Biometric recognition overcomes the disadvantages of traditional password- or token-based authentication; for example, passwords can be forgotten or guessed and tokens can be stolen or lost.

**Figure 1:**
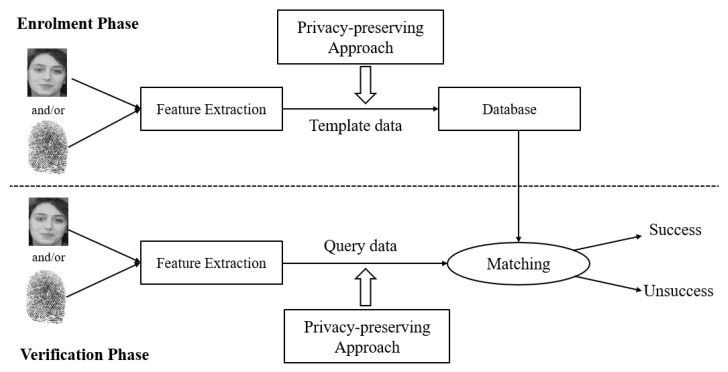
A privacy-preserving biometric system (adapted from [3]), with the facial image sourced from the ORL face database [4] and the fingerprint image from the FVC2002 fingerprint database [5].

A typical biometric system consists of two phases: the enrolment phase and the verification phase. In the enrolment phase, a user’s biometric data are extracted from his or her biometric sample (e.g., facial image or fingerprint scan) and stored in a database as a template. In the verification phase, the biometric data of a query, processed in the same way as in the enrolment phase, are compared or matched with the template to calculate a similarity score. If this score is greater than a pre-defined threshold, matching is successful; otherwise, matching is unsuccessful. Figure 1 shows a biometric system equipped with a privacy-preserving functionality (e.g., homomorphic encryption).

Despite the benefits brought by biometrics, biometric systems have their own weaknesses. Biometric data are uniquely linked to a person’s identity, and no two individuals in the world own exactly the same biometrics. Biometric data leaked in one application mean that they would be compromised in all other applications that depend on the same biometrics, which could lead to a data breach and identity fraud. With biometric security being a growing concern [6], researchers have developed a variety of biometric template protection techniques. Biometric template protection aims to secure the privacy and confidentiality of biometric template data while providing satisfactory recognition performance. Biometric template protection can be broadly divided into three categories—cancelable biometrics, biometric cryptosystems and homomorphic encryption (HE). These categories differ in their protection techniques, such as non-invertible transformation used by cancelable biometrics, key binding/generation employed in biometric cryptosystems and operation on ciphertext conducted by HE. The selection of the protection technique depends on specific applications and the desired level of security, as each category has its own properties, advantages and disadvantages, which are described below:

1. Cancelable biometrics: For security reasons, cancelable biometric systems do not store the original biometric data as templates. Instead, raw biometric data are transformed by a non-invertible transformation function in the enrolment phase, and the transformed data are stored in the database. Such a transformation is intentional and reproducible [7]. An essential property of cancelable biometrics is irreversibility, meaning that it should be computationally infeasible to retrieve the original biometric data from the transformed template [8]. In the verification phase, the same transformation is applied to the query data. Matching is performed in the transformed domain so that no original biometric data are divulged. If the stored (transformed) template is compromised, a new version can be generated by altering the transformation parameters. Cancelable biometrics is considered relatively simple and easy to implement.
2. Biometric cryptosystems: Bio-cryptosystems combine the benefits of biometrics and cryptography. In bio-cryptosystems, secret keys are either technically tied to or directly produced from biometric data. The original biometric data are encrypted by a secure sketch (e.g., Fuzzy Commitment [9], Fuzzy Vault [10] and PinSketch [11]) with helper data as the output. The helper data are generated by an irreversible cryptographic process so that it is difficult for adversaries to acquire the original biometric features from the helper data [12].
3. Homomorphic encryption (HE): HE tackles the data privacy issues by performing multiple operations on the encrypted data without any decryption [13]. Because the result of the HE computation remains encrypted and can only be decrypted by the data owners, confidentiality is kept and any third party can operate over the ciphertext without accessing the original plaintext [14].

HE is relatively new and promising compared to cancelable biometrics and bio-cryptosystems. It allows mathematical operations to be performed on encrypted biometric data without the need to decrypt them for authentication. In other words, biometric data can be encrypted and stored in databases without being decrypted during matching, thus preventing unauthorised access or privacy breaches. In addition, unlike cancelable biometrics, HE does not affect recognition accuracy. Overall, the application of HE in biometrics can protect the security and privacy of biometric data, while allowing for highly accurate identity verification.

### Organisation of This Work

This survey paper is organised into several sections that cover different aspects of HE and its applications to biometrics. The motivation and contributions of this work are stated in Section 2. In Section 3, different types of HE and specific HE schemes of each type are introduced and explained. In Section 4, the key aspects of the HE libraries that have been used or may potentially be used for biometric security are discussed and analysed. Various HE-based approaches to biometric security based on categories of different biometric traits are presented in Section 5, followed by discussions about the integration of HE with other technologies in Section 6. Challenges and future research directions are outlined in Section 7, before the paper concludes with a summary of the main findings in Section 8.

## 2. Motivation and Contributions

### 2.1. Motivation

A number of survey/review papers about HE and its implementation exist in the literature. To clarify and highlight the contributions of our work, we introduce and discuss the related survey papers below.

Munjal and Bhatia [15] gave a systematic review of homomorphic cryptosystems with their classification and evolution over time. Moreover, the authors reviewed homomorphic cryptosystems in healthcare. Marcolla et al. [13] provided a comprehensive review of HE from both the theoretical and practical points of view. This survey demonstrates the mathematical foundation required to understand fully homomorphic encryption (FHE). It also covers the design basis and security properties of FHE and the main FHE schemes based on mathematical problems. Thao et al. [14] compared and evaluated the performance of homomorphic cryptosystems using experimental results. This study describes all three families of HE, including the well-known schemes such as Ronald Rivest, Adi Shamir, and Leonard Adleman (RSA) and Paillier and their implementation specifications in widely used HE libraries (SEAL and HElib). Abreu and Pereira [16] overviewed the literature regarding privacy preservation for smart meters, focusing specifically on HE. The authors first described the notion of smart meters and the main concerns and oppositions inherent to their use. Then, two privacy-preserving approaches in two possible application scenarios were presented.

Gaytan et al. [17] analysed the basic concept, real-world implementation, cutting-edge methods, limitations, strengths, weaknesses and prospective applications of FHE. Given that the development of FHE in neural networks has shown significant progress in recent years, the authors focused on privacy-preserving homomorphic cryptosystems for neural networks, identifying current solutions, open problems, challenges, opportunities and future research. Chen et al. [18] used a web-based literature database and automated tools to describe the development of HE in machine learning (HEML). Several hot topics of HEML (e.g., cloud computing) were discussed in detail. The findings of this survey showed an increase in the number of articles published annually studying homomorphic cryptography and machine learning. Moreover, this survey found that half of the research contribution to HEML comes from China, the United States and India. Bansal [19] surveyed various HE techniques and classifications. The authors also reviewed applications of HE in cloud computing, e-voting and the IoT, its limitations and the scope of prospective growth. Wood et al. [20] gave an overview of FHE and its usage in medicine and bioinformatics. The authors presented the advanced notions behind FHE and its history, as well as the details of open-source implementations. The authors also expounded on the status of FHE in relation to privacy-preserving techniques in machine learning and bioinformatics and how these methods are applied in the field of cryptography.

It is not hard to see that, while the aforementioned survey papers reviewed the main HE methods and their implementation in applications such as cloud computing, bioinformatics and smart meters, none of them covered the topic of HE in biometrics. Biometric security is becoming an important and intense research topic. Compared to cancelable biometrics and biometric cryptosystems, HE is a relatively new and promising biometric template protection technique. It allows mathematical operations to be performed on encrypted biometric data without the need to decrypt them for authentication. Moreover, unlike cancelable biometrics, HE does not affect recognition accuracy. With these benefits, as an emerging biometric security technique, HE is desirable for protecting biometric data privacy. Therefore, we believe that a timely review of HE on biometric security is necessary and useful for advancing biometric-template-protection-related research.

### 2.2. Contributions

**Figure 2:**
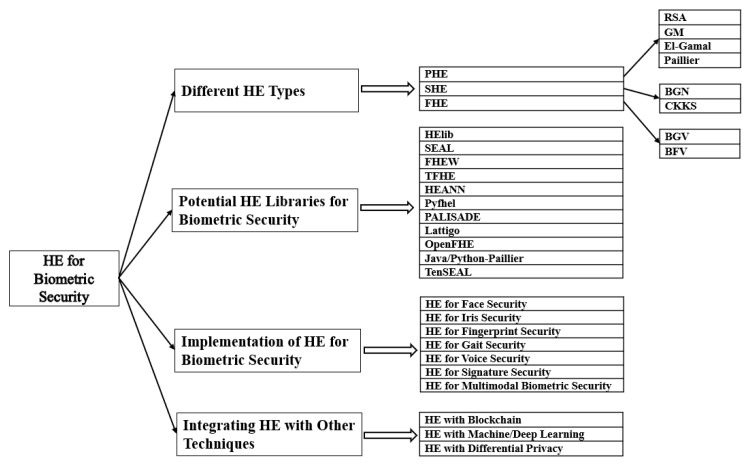
A taxonomy of the main points of knowledge in this paper.

A comprehensive and in-depth review of HE for privacy-preserving biometrics was conducted in this work to present the latest developments of HE methods that enhance the security of biometric data. The contributions of our work are summarised below:

1. To the best of our knowledge, this work is the first comprehensive review of HE for privacy-preserving biometrics. In this survey, state-of-the-art HE-based approaches to biometric security are discussed and analysed.
2. Various biometric-related HE methods were compared in terms of computational efficiency to provide readers a clear understanding of each method’s computing capacity.
3. Challenges and future research directions were set out to show potential pathways in the study of HE.
4. This review paper is a helpful reference for researchers working on privacy-preserving techniques in the area of biometric security (also known as biometric template protection). A taxonomy of the main points of knowledge in this review is given in Figure 2.

## 3. Homomorphic Encryption

A notion originated by Rivest, Adleman, and Dertouzos in 1978, HE allows calculation over encrypted data. This feature of HE is reflected in some well-known public key cryptosystems, such as the classic RSA [21] or El-Gamal [22], but it only works for one operation (addition or multiplication) and, sometimes, for a very limited number of two operations [23]. Several decades later, Gentry [24] in 2009 first proposed a public key encryption scheme capable of any kind of operation, namely fully homomorphic encryption (FHE).

**Figure 3:**
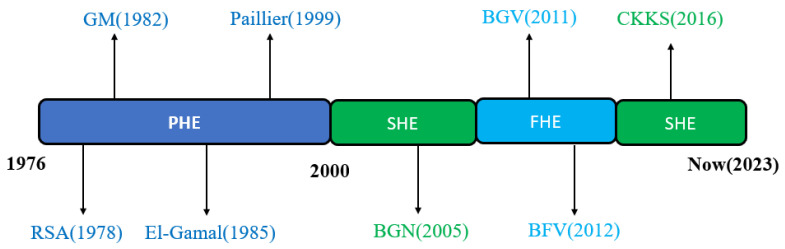
Timeline of some main schemes of each HE type (adapted from [14]).

HE allows for certain types of operations on ciphertexts without accessing the secret key. In addition, HE produces an encrypted result in which the decryption matches the computed result on the plaintext [25]. HE is classified based on a list of mathematical operations on encrypted data. The effectiveness and flexibility of HE are closely related to the number of operations on the list. HE schemes with a higher number of operations are considered more flexible, but have lower efficiency. Conversely, schemes with a smaller number of operations are less flexible, but more efficient [17]. Depending on the number of operations that are arbitrarily evaluated on the encrypted data, HE can be classified into different types, including FHE, partially homomorphic encryption (PHE) and somewhat homomorphic encryption (SHE). This section goes over different types of HE and the key features of the main HE schemes for each type. Interested readers can obtain more details of each scheme from the reference provided for each scheme. The timeline of some representative HE schemes in each type is displayed in Figure 3.

### 3.1. The Basics of HE

Building an HE scheme requires four steps [14]: key generation, encryption, decryption and homomorphic arithmetic operations (e.g., addition and multiplication). An encryption scheme is considered homomorphic [19] if it supports homomorphic addition and/or homomorphic multiplication, expressed by:

Homomorphic addition:
  (1) $$E\left(m_{1}\right)+E\left(m_{2}\right)=E\left(m_{1}+m_{2}\right),\;\forall m_{1},m_{2}\in M$$
Homomorphic multiplication:
  (2) $$E\left(m_{1}\right)*E\left(m_{2}\right)=E\left(m_{1}*m_{2}\right),\;\forall m_{1},m_{2}\in M$$
  where *E* represents an HE algorithm, *M* is the set of all possible messages, “+” denotes the addition operation and “*” the multiplication operation.

Different HE schemes involve different mathematical manipulations. Interested readers can refer to [14] for the details of a host of HE schemes (e.g., RSA [21], Paillier [26] and CKKS [27]). For the sake of demonstration, we give an example below to show the mathematical manipulations of a multiplicative homomorphic El-Gamal cryptosystem [28].

Key generation:

(1) A large prime number *p* is picked. (2) A generator $g\in Z_{p}^{*}$ is generated. (3) A random number *x*, where 1<x<p−1, is chosen, and then, $h=g^{x}mod\left(p\right)$ is calculated. (4) (p,g,h) forms the public key, while *x* is the secret key.

Encryption:

(1) A random number $k\in Z_{p}^{*}$ is chosen. (2) Given a message m∈M, its ciphertext is $E\left(m\right)=\left(a,b\right)=(g^{k}mod\left(p\right),mh^{k}mod\left(p\right))$.

Decryption:

For the ciphertext c=(a,b), after decryption, the original message *m* is derived as: $D\left(c\right)=D\left(a,b\right)=\frac{b}{a^{x}}mod\left(p\right)=m$.

Homomorphic multiplication:

$E\left(m_{1}\right)*E\left(m_{2}\right)=\left(g^{k1},m_{1}h^{k1}\right)\left(g^{k2},m_{2}h^{k2}\right)=\left(g^{k1+k2},m_{1}m_{2}h^{k1+k2}\right)=E\left(m_{1}*m_{2}\right)$

### 3.2. Partially Homomorphic Encryption

PHE allows an infinite number of operations of one type. For instance, additive HE allows an unlimited number of additions, but does not allow multiplication [17]. Below is a selection of the main PHE schemes:

- RSA [21]: Inspired by the Diffie–Hellmann key exchange problem [29], RSA was proposed in 1978. RSA is one of the first public key encryption methods for securing communication on the Internet. According to [17], RSA is considered the first multiplicative PHE.
- GM [30]: GM is the first probabilistic public key encryption scheme proposed by Goldwasser and Micali. The GM cryptosystem is based on the hardness of the quadratic residuosity problem.
- El-Gamal [22]: Being a multiplicative PHE scheme, the El-Gamal algorithm was derived from Diffie–Hellmann key exchange. Its security is based on the difficult mathematical problem known as the decisional discrete logarithm problem [14].
- Paillier [26]: Paillier is another probabilistic public key encryption scheme based on the composite residuosity problem [14], similar to the quadratic residuosity problem in GM. The Paillier scheme is homomorphic over addition and several extra basic operations on plaintexts.

### 3.3. Somewhat Homomorphic Encryption

SHE supports a predefined number of homomorphic operations, with the restriction on the number of permitted operations. Every operation adds to the underlying noise, so its proper evaluation relies only on performing a limited number of operations. When noise exceeds a certain threshold, the decryption of messages fails [17]. The key features of two main SHE schemes are introduced below:

- BGN [31]: Developed by Dan Boneh, Eu-Jin Goh and Kobbi Nissim, the BGN scheme was the first to support the addition and multiplication of ciphertexts with a constant size. It allows for any number of additions and a single multiplication operation on a ciphertext of a specified length. The homomorphic property of BGN allows users to evaluate multi-variate polynomials of a total degree of two given the encrypted inputs. The security of BGN is achieved under the assumption of the subgroup decision problem [17].
- CKKS [27]: Proposed by Jung Cheon, Andrey Kim, Miran Kim and Yongsoo Song, the CKKS scheme permits approximate addition and multiplication over ciphertexts whose plaintexts can be vectors of real or complex values. Since many HE schemes only work on binary or integer values, this feature of CKKS has attracted many researchers’ attention [14].

### 3.4. Fully Homomorphic Encryption

For FHE, there is no limit to the number of operations that can be undertaken [32]. The inherent characteristic of HE is that, each time a homomorphic operation is performed, the errors increase [13]. As a result, after a certain number of multiplications or additions, ciphertexts cannot be decrypted correctly because of the growth in the error. To address this issue, Gentry [24] introduced a technique, known as bootstrapping, which converts a scheme that is not fully homomorphic (e.g., SHE) into one that is fully homomorphic. In other words, FHE is built on a bootstrappable SHE. Two main FHE schemes are described below:

- BGV [33]: This scheme was a credit to Zvika Brakerski, Craig Gentry and Vinod Vaikuntanathan based on learning with error (LWE) or ring-LWE (RLWE) [33], without Gentry’s bootstrapping procedure [24]. Considered one of the hardest problems, which can be addressed in polynomial time, LWE has been intensively studied to build postquantum cryptographic solutions. As an algebraic variant of LWE, RLWE was put forth to have more efficient real-world applications with stronger security.
- BFV [34]: Considering the complexity and efficiency issues of FHE, Brakerski proposed several LWE-based FHE schemes, including Brakerski’s scale-invariant scheme [35]. BFV is the Fan–Vercauteren variant of Brakerski’s scale-invariant scheme [35]. It modifies the LWE setting in [35] to be RLWE. Using a simple modulus switching trick, BFV is more efficient than Brakerski’s scale-invariant scheme [35] according to [14]. The security of BFV-type cryptosystems is based on the RLWE problem.

### 3.5. Possible Attacks on HE Systems

Although HE can provide robust security, it is not exempt from attacks. A number of attacks can be initiated against HE systems, so it is vital to examine these attacks carefully before reviewing the application of HE to biometrics:

- Side-channel attacks [36]: Side-channel attacks assume that an adversary has access to some information about the secret key of the encryption algorithm. For example, the adversary launches timing attacks [37] that take advantage of the time a system spends on calculations while the encryption/decryption algorithm is being executed. Side-channel attacks are especially troublesome for HE as the encryption/decryption process involves a complex computation, which may leave a trace of information that can be exploited. A desirable security requirement for HE schemes is to have resistance to such attacks, often called leakage resilience, meaning that semantic security should not be breached, even in the case of side-channel attacks.
- Black box attacks [38]: A black box attack on HE takes place when an adversary gains access to the encrypted data and manipulates them, but the adversary has no access to the secret key. The adversary’s objective is to obtain information about the plaintext data by examining the output of the homomorphic operation. Through randomised encoding, such as adding a random value to the plaintext before encryption, black box attacks can be tackled.
- Lattice attacks [39]: A lattice attack is a form of attack exploiting the vulnerabilities in lattice structures to restore the secret key in a lattice-based cryptosystem. This type of attack can be used to target some lattice-based HE schemes. For example, it was shown in [39] that, under certain parameter settings, an attacker could directly derive the plaintext from the ciphertext and public key even without using the secret key of the lattice-based FHE.
- Other attacks: Other attacks that target HE include attacks on broadcast encryption [40], chosen ciphertext key recovery attacks [40], chosen related plaintext attacks [40], decoding attacks on LWE [36] and reaction attacks [41].

## 4. Potential HE Libraries for Biometric Security

HE libraries play a pivotal role in helping researchers and professionals implement HE in many applications including biometrics. The efficiency of these applications has been greatly improved by the evolution and optimisation of HE libraries over the past few years [16]. In this section, HE libraries [14,17] that have been adopted or will potentially be implemented for biometric security are summarised in Table 1 and discussed below:

- SEAL [42]: SEAL stands for Simple Encrypted Arithmetic Library. Developed by Microsoft’s Cryptography and Privacy Research Group, SEAL was first released in 2015 for the specific purpose of making available a well-designed and recorded HE library. SEAL suits both experts and non-experts having little or no background in cryptography. Recent releases of Microsoft SEAL have incorporated a diverse range of HE schemes, such as BGV, BFV and CKKS. SEAL is implemented in C++ and going through active development in other languages (e.g., C#, Python and JavaScript). For example, a Python version of SEAL is available, called PySEAL [43].
- HElib [44]: HElib stands for Homomorphic-Encryption Library. Released in 2013, HElib is the first open-source library that implements HE. Developed in C++, HElib specialises in the efficient use of BGV, CKKS and ciphertext packing schemes, as well as Gentry–Halevi–Smart optimisations. After releasing the first build of HElib, the authors made algorithmic improvements, such as high-speed homomorphic linear transformations, enabling HElib to be much faster than the previous builds.
- TFHE [45]: TFHE refers to Faster Fully Homomorphic Encryption. Released in about 2016, TFHE is an open-source library that persists in the ring variant of the Gentry–Sahai–Waters (GSW) scheme [46].Developed in C/C++, TFHE is a very fast door-by-door bootstrap program with no restrictions on the number of gates or their composition.
- FHEW [47]: FHEW is the acronym for Fastest Homomorphic Encryption. Built on a fully homomorphic encryption scheme [48], the first version of FHEW was released in about 2015. Written in C, this library offers symmetric encryption to encrypt/decrypt single-bit messages and supports homomorphic assessment of encrypted data using a public key for arbitrary Boolean circuits.
- HEANN [27]: HEAAN stands for Homomorphic Encryption for Arithmetic of Approximate Numbers. Developed in C++ and first released in 2016, HEAAN is a library that supports fixed-point arithmetic and CKKS.
- PALISADE [49]: Developed in an open-source C++ project, PALISADE was first released in 2019. An effective realisation of the lattice cryptography build block, PALISADE supports a number of HE schemes (e.g., BGV, BFV and CKKS). It also allows multiparty extensions of selected HE schemes and relevant primitives of cryptography, such as digital signature techniques, proxy re-encryption and program obfuscation.
- Lattigo [50]: Implemented in Go [51] and released in 2019, Lattigo is a lattice-based encryption library designed to support HE schemes (e.g., BFV, BGV and CKKS) in distributed systems and microservice architectures. It implements RLWE-based HE primitives and multiparty-homomorphic-encryption-based security algorithms.
- Pyfhel [52]: Pyfhel stands for Python For Homomorphic Encryption Library. First released in 2018, Pyfhel enables some HE operations in Python, such as addition, multiplication, exponentiation or scalar products. This library is suitable for both simple HE demonstrations and complicated problems such as machine learning algorithms. Pyfhel was built using Python and Cython on top of Abstraction Homomorphic Encryption Library (Afhel) in C++.
- OpenFHE [53]: Written in C++, OpenFHE is an open-source FHE software library. It combines design concepts from the FHE projects PALISADE, HElib and HEAAN and also includes new design ideas. OpenFHE has efficient implementations of common FHE schemes [54], such as BFV, BGV and CKKS.
- Python-Paillier [55]: Written in Python, Python-Paillier was designed, built and supported by CSIRO’s Data61. This library makes it possible for encrypted numbers to be added together, multiplied by a non-encrypted scalar or added to a non-encrypted scalar.
- Java-Paillier [56]: Java-Paillier is a Java implementation of Paillier PHE.
- TenSEAL [57]: TenSEAL is a library for cryptographic tensor computation using HE. It allows tensors to be converted directly from popular machine learning frameworks (e.g., PyTorch and Tensorflow) into encrypted versions. As such, it equips classical machine learning frameworks with HE capabilities. TenSEAL is the implementation of the CKKS program in Microsoft SEAL. It supports both C++ and Python.

**Table 1:** HE libraries for biometric security (adapted from [13,15]).

HE Library	Year Released	HE Schemes Supported	Development Language
HElib [44]	2013	BGV and CKKS	C++
Python-Paillier [55]	2013	Paillier	Python
Java-Paillier [56]	-	Paillier	Java
SEAL [44]	2015	BGV, BFV and CKKS	C++
FHEW [47]	2015	-	C
TFHE [45]	2016	Ring variant of GSW	C/C++
HEANN [27]	2016	CKKS	C++
Pyfhel [52]	2018	BGV, BFV and CKKS	Python and Cython
PALISADE [49]	2019	BGV, BFV and CKKS	C++
Lattigo [50]	2019	BGV, BFV and CKKS	Go
TenSEAL [57]	2021	CKKS	C++ or Python
OpenFHE [53]	2022	BGV, BFV and CKKS	C++

## 5. HE-Based Approaches to Biometric Security

In this section, HE-based approaches to biometric security and their performance are discussed and compared in terms of computational efficiency. We categorise these approaches by different biometric traits under protection.

### 5.1. HE-Based Approaches to Face Security

HE-based approaches to face security deal with the encryption of facial recognition data using HE algorithms, allowing calculations to be carried out on encrypted data with no need to decrypt them first and, thus, protecting sensitive facial data. Table 2 compares different HE approaches to face security.

Shahreza et al. [58] proposed a hybrid solution to securing face templates by combining the cancelable biometric (CB) technique and HE. Since the protected templates are irreversible even in the case of a compromised secret key (often referred to as the fully compromised case), using CB prior to HE strengthens the security and privacy of the whole system and reduces template dimensions, which accelerates the computation of ciphertexts. Román et al. [59] used public key encryption and HE to protect facial data. The experimental results showed that recognition performance is retained after protection. The proposed method also renders size-reduced protected templates and keys and a fast execution time compared to other lattice-based HE schemes. Bauspieß et al. [60] developed an improved coefficient-packing-based FHE method to secure face templates. Capable of feature dimensionality reduction, the proposed method streamlines computations. The experimental evaluation over a public face database showed that efficient face recognition in the cryptographic domain (up to a 1.6% reduction in computing time) can be achieved on off-the-shelf hardware with no loss in recognition accuracy.

Building on the M-tree data structure and symmetric HE, Yang et al. [61] proposed privacy-preserving biometric identification over the cloud, calling it MASK. With recognition accuracy maintained, MASK ensures the privacy of users’ recognition requests (e.g., face recognition) and service providers’ datasets, while greatly reducing cloud servers’ computing cost on biometric dataset searching. Pradel et al. [62] presented an FHE-based privacy-preserving biometric authentication scheme, in which users’ biometric samples (e.g., face images) are collected by a local device, but matched through a remote server on encrypted data entirely. By this means, users’ sensitive biometric data are kept private with authentication carried out by the server.

Drozdowski et al. [63] reduced the computational overheads associated with face recognition transactions without dropping recognition performance. Through the seamless integration of template protection with open-source HE libraries, the proposed method guaranteed the irreversibility, unlinkability and renewability of the protected biometric data. Jindal et al. [64] designed an FHE-based biometric template protection method, which is both computationally efficient and practical. In contrast to most existing HE schemes, the proposed method supports the manipulation of real-valued biometric feature vectors without quantisation so that they can be packed into a single ciphertext. Drozdowski et al. [65] proposed a system framework that allows face recognition in the cryptographic domain. This framework offers and assesses the implementation of HE schemes. Biometric-related concerns and challenges, as well as future research pathways were put forward.

In this section, HE-based approaches to face security are reviewed, which shows the potential of using HE to secure face biometric data and protect user privacy. It can be seen from Table 2 that most of the HE-based approaches rely on SEAL to handle either binary-valued or floating-point data. The data size reported in these approaches ranges from 32 to 512 bits for binary data and 128 to 512 dimensions for real-valued data. Although different hardware configurations affect system performance in terms of optimal computing time, it is necessary to improve system efficiency and scalability and make HE systems resistant to attacks (e.g., side-channel attacks and black box attacks).

### 5.2. HE-Based Approaches to Iris Security

In iris recognition, cameras are used to capture high-resolution images of the iris, from which unique features are extracted, such as the texture, shape and pattern of the iris. As one of the most-accurate biometric authentication modalities, there is ongoing research in protecting iris data [69]. A comparison of HE-based approaches to iris security is given in Table 3.

Morampudi et al. [70] proposed a secure and verifiable classification-based iris authentication system, named SvaS, with FHE on a malicious cloud server. SvaS aims at privacy-preserving training and privacy-preserving classification of nearest-neighbour and multiclass perceptron models. The BFV scheme [34] provides security protection to iris templates. In this scheme, the ensemble verification vector is responsible for verifying the correctness of the computed classification results. Song et al. [71] introduced an iris-based ciphertext authentication system using FHE and the fuzzy vault. Authentication is performed with no decryption of iris templates whose homomorphic ciphertexts are stored in the database, so there is no disclosure about the iris templates. Furthermore, the proposed system eliminates the need for trust centre authentication as authentication is conducted directly on the server side using a one-time message authentication code. Morampudi et al. [76] designed FHE-based iris authentication for protecting template data and restricting data leakage. This method generates a rotation-invariant iris code to enhance recognition accuracy and reduces computing time via batch processing. The experimental results demonstrated that the proposed method can be practically implemented without a loss of accuracy while preserving the privacy of iris templates.

Torres et al. [72] conducted a study on identifying the effectiveness of lattice-based FHE for privacy preservation of biometric data in authentication systems. Implemented on an iris authentication system and according to the experimental results, the FHE approach showed protection of the privacy of iris data. The study also found that the main issue with the FHE approach is recognition performance and ciphertext size. Torres et al. [77] investigated the efficacy of FHE in biometric systems. Lattice-based FHE was applied to iris authentication systems for preserving the privacy of iris feature data. The authors also conducted a security analysis on authentication and privacy preservation. Luo et al. [74] proposed an anonymous biometric access control system that uses biometric data (e.g., iris data) to validate a user’s membership without the knowledge of the user’s true identity. The authors adopted HE to safeguard iris data and developed a secure similarity search algorithm to perform validation anonymously. The proposed system helps to protect the privacy of authorised users and reject impostors.

In this section, HE-based approaches to iris security are reviewed. We found that the number of HE-based research articles for iris security is slightly lower than that for face security, but higher than that for fingerprint, gait, voice or signature security. It can be observed from Table 3 that the data type of these studies is largely binary, and the data size ranges from 640 to 9600 bits.

### 5.3. HE-Based Approaches to Fingerprint Security

Fingerprints are one of the most-widely used biometric traits. Fingerprint recognition utilises the unique pattern of the ridges and valleys on a person’s fingerprints for identity authentication [78]. HE-based methods for fingerprint security are discussed below.

Yang et al. [79] proposed an HE-based fingerprint authentication system for access control and protecting sensitive fingerprint template data. Due to the use of HE, fingerprint matching takes place in the encrypted domain, making it difficult for adversaries to gain access to the original fingerprint template in the absence of the private key. The authors also analysed the trade-off between computing time and recognition accuracy. Barni et al. [80] introduced a privacy-preserving fingerprint recognition system. In this system, users’ fingerprint samples are collected at the client side and encrypted to form Fingercode templates. The system handles matching tasks in the cryptographic domain using HE and Fingercode templates.

Despite active research on non-HE approaches to fingerprint security, such as cancelable fingerprint templates and bio-cryptographic fingerprint systems, there is a lack of momentum for HE studies on fingerprint data protection compared to HE-based approaches to face security and iris security. Therefore, more research on HE methods for fingerprint security is in demand, given that the fingerprint is one of the most-popular biometric traits extensively in use and takes the largest market share in real-world applications.

### 5.4. HE-Based Approaches to Gait Security

Each person has a distinctive gait, which can be used to distinguish them. Gait recognition utilises the way a person walks to recognise them. Lin et al. [81] proposed HE-based gait recognition to protect sensitive gait feature data. Different from fingerprint or face data, which are time-independent, gait features are time-dependent and continuous. The authors modified a convolutional neural network (CNN) and combined it with FHE to handle encrypted gait data. Table 4 reports the comparison of HE-based approaches to fingerprint security and gait security.

### 5.5. HE-Based Approaches to Voice Security

Voice recognition [82], also referred to as speaker recognition, authenticates individuals according to the unique characteristics of a person’s voice, such as intonation, tone of voice and accent. Rahulamathavan [83] redesigned the back-end of speaker verification systems to alleviate the privacy concerns of speech features. Based on the Newton–Raphson method, the authors proposed a solution to addressing the limitation of CKKS (i.e., computing the inverse square root of encrypted numbers), yielding negligible loss in recognition accuracy with reduced multiplication depth. Nautsch et al. [84] introduced two architectures for voice recognition with two covariance comparators. In both architectures, the privacy of biometric data is preserved by extending the HE scheme of the cosine similarity comparison. In addition, biometric service providers can provide the same comparison module using different key pairs for different biometric service operators.

### 5.6. HE-Based Approaches to Signature Security

Signature recognition makes use of the unique characteristics of a person’s signature to identify them [85]. In signature recognition systems, a digital pen or touchpad is used to capture users’ signatures, which are processed to extract distinctive features, such as the order of strokes, stress and writing speed. Barrero et al. [86] proposed HE-based biometric template protection, in which only encrypted data are processed and templates are of a fixed length. In a completely repeatable experimental framework, the authors analysed different distance measures in the scenario of online signatures, showing that all requirements for biometric template protection (e.g., irreversibility, unlinkability and renewability) are met without compromising recognition performance and with a low computational cost. Table 5 gives the comparison of HE-based approaches to voice security and signature security.

### 5.7. HE-Based Approaches to Multimodal Biometric Security

Multimodal biometric systems use multiple biometric modalities to identify and authenticate individuals [87]. Multimodal biometric systems are considered more robust than their unimodal counterparts. With combinations of different modalities, HE-based approaches to multimodal biometric security are discussed below and compared in Table 6.

#### 5.7.1. Iris and Fingerprint

Vallabhadas et al. [88] studied biometric template protection with two biometric traits (i.e., iris and fingerprint). Feature vectors formed from the extracted features of the two traits are fused and transformed by local random projection to create revocable and unlinkable templates, to which FHE is applied for privacy protection purposes. Salem et al. [89] proposed a cloud-based multi-party privacy-preserving biometric recognition system using HE. By taking advantage of transfer learning, training sensitive biometric data (e.g., the fusion of iris and fingerprint data) are relinquished, and a pre-trained deep neural network serves as a feature extractor, performing biometric verification and liveness detection tasks. As there is no need to train and decrypt sensitive biometric data, the proposed system guarantees privacy and is highly scalable.

#### 5.7.2. Signature and Fingerprint

Barrero et al. [90] proposed an HE-based general framework for the protection of multi-biometric templates (e.g., signature and fingerprint templates). The authors gave in-depth analyses of three levels of fusion (i.e., feature, score and decision levels). Moreover, no decryption is required during the verification phase even though all processes are performed in the encrypted domain, resulting in efficient verification that is implementable in real-time.

#### 5.7.3. Face and Voice

Sperling et al. [91] presented a non-interactive end-to-end approach to secure fusion of biometric templates using FHE. A pair of face and voice feature vectors encrypted by FHE are first fused through concatenation. Then, the dimension of the fused feature vectors is reduced through learned linear projection, followed by feature scale normalisation and matching score calculation.

This section discusses HE-based methods for multimodal biometrics, combining a widely used biometric trait, such as fingerprint or face, with one or more other traits to achieve better recognition accuracy and/or security. However, the use of multimodal biometrics introduces operational overheads, such as additional sensors and more feature extraction, processing and matching costs. As such, the effectiveness of these approaches depends on the individual design and application requirements, which often require a trade-off between recognition performance and resource allocation.

### 5.8. HE-Based Approaches to the Security of Non-Specific Biometric Modalities

Some HE-based approaches can be applied to any biometric modality as long as the extracted feature data are in the format of binary vectors of a fixed length. Karabat et al. [92] presented a generic biometric authentication and template protection system, whose feature extraction yields binary templates of a fixed size. The proposed system (named THRIVE) consists of a registration and authentication protocol based on threshold HE, in which a private key is to be shared between the user and the verifier. In THRIVE, only encrypted binary templates are stored in the database and verified by homomorphic random templates, so that the original templates are not disclosed during the verification stage. Thanks to the underlying threshold HE scheme, a hostile database owner cannot fully decrypt the encrypted templates of users in the database. Mandal et al. [93] built a cryptographically secure system using the HE scheme proposed by Brakerski and Vaikuntanathan [94]. The authors designed a challenge–response authentication mechanism and a decentralised architecture in which calculation and authentication are separated. The proposed system can protect any binary-type biometric templates. Yasuda et al. [95] studied HE-based privacy-preserving biometric authentication. The authors proposed an effective method to calculate the Hamming distance of encrypted data using ideal lattice-based HE. The experimental results showed that the proposed method achieved fast recognition performance and reduced the ciphertext size compared to the existing related work.

## 6. Integrating HE with Other Technologies for Biometric Security

Integrating HE with other technologies can bring extra benefits to biometric authentication, such as improved security and recognition accuracy. In this section, we review current works that integrate HE with other innovative technologies, including blockchain, machine learning/deep learning and differential privacy.

### 6.1. HE with Blockchain

Blockchain is an advanced technology that delivers the service of decentralised data storage and the capability to record and protect transactions using cryptography [96]. All the nodes involved in the blockchain know every transaction that occurs in the blockchain [97]. Integrating HE and blockchain technology provides a powerful combination for biometric security, allowing sensitive biometric data to be processed without compromising security. For example, Kumar et al. [75] proposed a multi-instance iris authentication system to counter malicious attacks on transmission channels and untrusted servers. The proposed system utilises El-Gamal to encrypt iris templates. Smart contracts operating on the blockchain ensure the integrity of iris templates and match results. The proposed system also overcomes the drawback of blockchain use for biometrics (e.g., the privacy issue and costly storage).

### 6.2. HE with Machine/Deep Learning

With machine/deep learning technology entering many industries, as well as people’s lives, privacy and security concerns arise from system users, operators and administrators. Since CNNs are extensively employed to handle complicated visual tasks, integrating HE with machine/deep learning offers strong privacy protection for biometric systems. Wingarz et al. [66] detailed the steps to create a privacy-preserving CNN and analysed its applicability and scalability in the real world. In this context, a homomorphically encrypted neural network was implemented for face recognition. The simulation results showed that running a CNN on homomorphically encrypted inputs achieved the same recognition accuracy as in a conventional CNN case. Sun et al. [67] proposed a secure face recognition system based on HE to avoid facial data operated in plaintext. Face image features, extracted by a deep learning (DL) model, are wrapped into ciphertexts using HE and batch processing.

Authentication is carried out without decrypting facial data, which reduces the risk of data leakage. It should be noted that, even though the operation on facial feature data occurs in the encrypted domain, attacks such as side-channel attacks, black box attacks and lattice attacks (as introduced in Section 3.5) can be launched. Therefore, it is important to identify security vulnerabilities and improve the security strength of HE systems.

Tamiya et al. [68] designed face template protection using HE and DL. The authors exploited a DL-based feature extraction algorithm and an HE scheme to encrypt integers. The message-packing method adopted in the proposed system allows the squared Euclidean distance between facial features to be computed through a single homomorphic multiplication.

### 6.3. HE with Differential Privacy

With applications in many fields (e.g., statistics and data analysis), differential privacy (DP) provides a robust protocol for privacy preservation. The basic idea of DP is to protect the privacy of individual data points by incorporating “noise” in the data so that nobody’s data can be distinguished from any other individual’s data [98]. Combining HE and DP in biometric systems renders an effective tool for protecting the privacy of biometric data, while permitting sophisticated data manipulation and analysis. Raisaro et al. [99] designed and deployed an efficient privacy-preserving explorer for genomic cohorts in a real-world operational environment. Advanced privacy-enhancing techniques (DP and HE) are used to outsource and explore massive amounts of genomic and clinical data, with HE protecting the confidentiality of patients’ genomic data against unauthorised access and DP preventing re-identification attacks. The proposed system can securely and simultaneously calculate simple statistics for more than 3000 encrypted genetic variants for a cohort of 5000 people within 5 s using commercial hardware.

## 7. Challenges and Future Research Directions

### 7.1. Challenges

The study of biometric data privacy protection has attracted considerable attention from academic researchers and industry professionals due to the wide applications of biometrics. HE is an emerging technology applied to biometrics. It is still under development compared to other mature techniques, such as cancelable biometrics and biometric cryptosystems. Therefore, more work is required to improve its capability and overcome its shortcomings in the application of HE. Below are some of the challenges that need addressing so as to ensure the success of HE for biometric security:

- Limited operations/functionalities: Many HE schemes can only carry out specific calculations, such as addition and multiplication. However, biometric systems (e.g., face recognition and fingerprint authentication) may need HE schemes to be able to handle more advanced mathematical operations, such as convolutions, Fourier transforms and exponential and logarithmic operations on encrypted data. Although some computations (e.g., loss function calculation in the encrypted domain with privacy-preserving DL [100]) are adaptable to basic operations such as addition and multiplication, efficiency and accuracy may be compromised.
- Potential vulnerabilities: The implementation of HE in biometrics provides additional privacy protection for the storage and comparison of biometric data, but HE schemes are relatively new and have not been extensively studied for their counter-attack ability. When implemented in biometric systems in practice, HE schemes may suffer from potential attacks and show vulnerabilities. For example, research reveals that HE schemes can be exposed to attacks such as decoding attacks, dual attacks, side-channel attacks [36], key-recovery attack [101] and reaction attacks [41]. Privacy issues also arise during data manipulation in HE-based systems. As noted in [102], the convenience of direct manipulation on encrypted data makes it hard for HE-based systems to track intermediate computations. This increases the possibility of a malicious attack where sensitive biometric data may be leaked from a client or server compromised by an adversary.
- Technical complexity: Implementing HE schemes in biometrics can be technically complex and demands a sound understanding of the underlying mathematics and even encryption itself [24]. Since many industry professionals, especially novices, do not have the required level of relevant mathematical knowledge about HE, it would be challenging for them to implement HE schemes in biometric systems. For this reason, academics and researchers should develop easy-to-use HE schemes and libraries to facilitate the implementation of HE in biometric applications.
- Computational complexity: HE schemes are computationally intensive and can be resource-intensive as well, making real-time HE applications in biometrics a challenge. For example, as shown in Section 5, the computing time (e.g.,transactions) of some HE-based biometric systems could take more than 10 s (see, e.g., Pradel et al. [62], Torres et al. [72], Luo et al. [74] and Barni et al. [80]). Since practical biometric applications (e.g., access control) may require timely responses, the computationally intensive nature of HE schemes is likely to cause too much delay or latency, thus making it challenging to use HE in large-scale or time-pressing biometric systems where low latency is required.
- Performance trade-off: Allowing computations to be conducted on encrypted data without decrypting them makes HE a powerful tool, but there are substantial overheads associated with HE applications in biometrics. For example, the ciphertext size is typically larger than the plaintext size, so encrypted biometric data require more computational resources and storage space [79]. Furthermore, operations on encrypted data can be time-consuming. In order to speed up the encryption and decryption operations, advanced and specialised hardware platforms have to be chosen for HE schemes applied to biometric security. Although it is appealing for HE-based privacy-preserving biometric systems to have good recognition performance, high efficiency and strong security, they are likely competing criteria, which may entail a performance trade-off.
- Key management: The security of HE-based biometric systems heavily relies on the management of cryptographic keys. Usually, the public key is for encryption purposes, whereas the private key is for decryption purposes. The management of the private key is critical for securing the confidentiality of encrypted biometric data. Lee et al. [103] introduced the concept of hierarchical Galois key generation for HE to relieve the burden of clients and the server running BFV and CKKS schemes. Unfortunately, most of the existing key management methods are developed for general HE applications rather than HE-based biometrics. Therefore, it is imperative to specifically design key management methods suitable for HE-based biometric systems.

### 7.2. Future Research Directions

Although applying HE to privacy-preserving biometric identification has made significant progress, it still faces unsolved issues, such as high computational complexity, low efficiency and inadequate deployment in the real world. Further research is needed to make HE-related encryption, decryption and matching processes more efficient and practically implementable. If neural networks are used, efficiency becomes demanding when training and evaluating complex neural networks on encrypted data or training cryptographic neural networks on plaintext data. Several future research directions are highlighted below:

- Development of efficient HE schemes: There is a demand to devise new HE solutions that are faster and require fewer resources or to develop ways of optimising existing HE schemes for biometric applications.
- Combination of HE with other techniques: In order to strengthen security, it is worth investigating the combination of HE with other privacy-enhancing techniques, such as blockchain and DP. Furthermore, finding stable and high-quality feature-learning approaches will improve the recognition accuracy of HE-based biometric systems.
- Development of HE solutions that can handle sophisticated operations: This involves developing HE schemes capable of more advanced mathematical operations (e.g., convolution and Fourier transforms), as they may be required by the training, feature transformation and matching procedures of biometric systems.
- Counter-attack research: With an increasing risk of attacks on HE-based biometric authentication systems, it is of necessity to study how to defend various attacks and make HE schemes more robust.
- Practical implementation of HE schemes: Although the practicability of HE in real-world biometric applications is constrained by factors such as technical complexity and hefty computation, HE is a promising technique from the security point of view. When implementations are optimised and streamlined, HE solutions will be more effective and practical.

## 8. Conclusions

In conclusion, this survey paper provides an overview of the application of HE in biometrics. Specific HE schemes under different HE types were discussed, along with the availability and specifications of HE libraries that have been used or can be potentially used for biometrics. This paper presented various HE-based approaches to the security of face, fingerprint, iris and other biometrics. The integration of HE with other technologies was also discussed whilst highlighting the challenges and future research directions of HE in biometrics. Overall, this survey thoroughly reviewed the current state of HE for biometric security, which is of benefit to readers specialising in biometrics research.

## Figures and Tables

**Table 2:** Comparison of HE-based approaches to face security in terms of computational efficiency.

Scheme (Year)	HE Library	Trait (Database)	System/Hardware Specifications	Comparison on Data Type or Format, Data Size and Optimal Computing Time
Shahreza et al. [58] (2022)	SEAL	Face (ArcFace, ElasticFace and FaceNet)	Intel(R) Core(TM) i7-7700K CPU @ 4.20 GHz.	Data: binary-valued, 32 to 512 bits. Time: Encoding—1.19 ms. Comparison—23.14 ms. Decoding—0.38 ms.
Román et al. [59] (2022)	-	Face (FERET and LFW)	Intel Core i7-1165G7 laptop @ 2.80 GHz.	Data: binary-valued, 1.44 KB. Time: Key generation—1.27 ms. Encryption—3.04 ms. Comparison—0.88 ms.
Bauspieß et al. [60] (2022)	PALISADE	Face (VGGFace2)	Single core Intel i7-10750H processor of 2.60 GHz.	Data: binary-valued, 64 to 512 bits. Time: Single identification transaction—0.82 ms.
Yang et al. [61] (2021)	-	Face (dataset of University of Essex)	Intel Core i7-8750H CPU @ 2.1 GHz and 16 GB RAM.	Data: no information. Time: no information.
Pradel et al. [62] (2021)	TFHE	Face (-)	Ubuntu 20.04.1 LTS 64-bit machine with 8GB RAM and a quad-core Intel(R) Core(TM) i3-6100 CPU @ 3.70 GHz.	Data: binary-valued, 128 bits. Time: addition, subtraction and multiplication of two 128-bit feature vectors take 9 s, 30 s and 206 s, respectively.
Drozdowski et al. [63] (2021)	SEAL	Face (MORPH)	Linux Debian 10 and a commodity notebook of an Intel Core i7 2.7 GHz CPU with 16GB DDR4 RAM.	Data: no information. Time: Key generation—362 ms. Encryption/decryption—27 ms. Comparison—23 ms.
Jindal et al. [64] (2020)	-	Face (LFW, FEI and Georgia Tech)	Server with Intel Xeon Gold CPU clocked @ 2.4 Ghz with 64 GB RAM and 32 cores.	Data: real-valued, 128 dimensions. Time: Matching two encrypted face templates—2.83 ms.
Drozdowski et al. [65] (2019)	SEAL	Face (FERET)	Virtualised Linux environment and one 2.5 GHz CPU with 8 GB RAM.	Data: floating-point, 512 dimensions. Time: Encryption/decryption—2.5 ms. Computing the distance between two encrypted feature vectors—850 ms
Wingarz et al. [66] (2022)	SEAL	Face (Yale Face Database B)	Intel(R) Xeon(R) Gold 6130 CPU @ 2.10 GHz server with 256 GB RAM.	Data: no information. Time: Single image execution—0.2549 s.
Sun et al. [67] (2022)	SEAL	Face (LFW, IJB and CASIA)	Intel Core i7-6700HQ processor.	Data: binary-valued, 64 to 128 bits. Time: no information.
Tamiya et al. [68] (2021)	-	Face (FERET)	Ubuntu 18.04 machine with Intel Core i7-8700 3.2 GHz CPU and 16 GB DDR RAM.	Data: binary-valued, 128 to 2048 bits. Time: Total transaction—49.5 ms.

**Table 3:** Comparison of HE-based approaches to iris security in terms of computational efficiency.

Scheme (Year)	HE Library	Trait (Database)	System/Hardware Specification	Comparison on Data Type or Format, Data Size and Optimal Computing Time
Morampudi et al. [70] (2021)	-	Iris (CASIA-V 1.0, CASIA-V3-Interval, IITD and SDUMLA-HMT)	2.40 GHz Intel i7 processor with 16 GB RAM.	Data: binary-valued, 640 to 2560 bits. Time: Encryption—0.003 s. Decryption—0.0008 s. Similarity score calculation—1.19 s.
Song et al. [71] (2020)	SEAL	Iris (CASIA-Iris)	HP notebook with Intel Core i5-6200U processor.	Data: binary-valued, 2048 bits. Time: Encryption—104.5 ms. Decryption—231.6 ms.
Torres et al. [72] (2015)	Lattice-based cryptography library written in Java [73]	Iris (BATH)	Intel Core i7-3630QM @ 2.40 GHz with 16 GB RAM.	Data: binary-valued, 2048 bits. Time: Key generation—26.649 s. Encryption—3.8 min. Decryption plus comparison—0.49 s.
Luo et al. [74] (2009)	Paillier cryptosystem	Iris (CASIA-Iris)	Linux machine with AMD Athlon 64, 2.4 GHz and 2 GB memory.	Data: binary-valued, 9600 bits. Time: Encryption—289.922 s. Decryption—17.946 s. Similarity search with threshold comparison—42.189 s.
Kumar et al. [75] (2020)	-	Iris (CASIA-V3-Interval, IITD and SDUMLA-HMT)	Intel Core i5 processor of 2.50 GHz and 16 GB RAM.	Data: binary-valued, 1280 to 2560 bits. Time: Distance calculation—6.0254 s.

**Table 4:** Comparison of HE-based approaches to fingerprint security and gait security in terms of computational efficiency.

Scheme (Year)	HE Library	Trait (Database)	System/Hardware Specification	Comparison on Data Type or Format, Data Size and Optimal Computing Time
Yang et al. [79] (2020)	Python-Paillier	Fingerprint (FVC2002 DB2)	Desktop with AMD processor AMD FX-8370 8-Core Processor @ 4.01 GHz with 24GB RAM.	Data: binary-valued, 300 to 600 bits. Time: Key generation—1.7 s. Encryption of 300 bits—2.1 min. Comparison—3 s.
Barni et al. [80] (2010)	-	Fingerprint (dataset from Microtechnology)	PC with 2.4 GHz CPU and 4 GB RAM.	Data: floating point, 640 dimensions. Time: Single identification transaction—37.43 s.
Lin et al. [81] (2022)	HElib	Gait (CASIA-B)	-	Data: no information. Time: no information.

**Table 5:** Comparison of HE-based approaches to voice security and signature security in terms of computational efficiency.

Scheme (Year)	HE Library	Trait (Database)	System/Hardware Specification	Comparison on Data Type or Format, Data Size and Optimal Computing Time
Rahulamathavan [83] (2022)	TenSEAL and SEAL	Voice (TIMIT)	Razor laptop with 16 GB RAM and 6 cores (12 CPUs) @ 4.1 GHz (max).	Data: binary-valued, 1024 to 32,768 bits. Time: Encryption—between 11 ms and 55 ms. Decryption—between 1 ms and 12 ms. Verification—1.3 s.
Nautsch et al. [84] (2018)	Python-Paillier	Voice (NIST Machine Learning Challenge Phase III Database)	-	Data: double floating-point, 600 dimensions. Time: no information.
Barrero et al. [86] (2016)	Java-Paillier	Signature (DS2 BioSecure Multimodal database)	Intel Core i7 with four 2.67 GHz cores	Data: real-valued, 200 dimensions. Time: Single comparison bout—0.1 ms.

**Table 6:** Comparison of HE-based approaches to multimodal biometric security in terms of computational efficiency.

Scheme (Year)	HE Library	Trait (Database)	System/Hardware Specification	Comparison on Data Type or Format, Data Size and Optimal Computing Time
Vallabhadas et al. [88] (2022)	SEAL	Fingerprint and iris (Children Multimodal Biometric Database)	-	Data: binary-valued, 2560 bits (iris) + 1024 bits (fingerprint). Time: no information.
Salem et al. [89] (2018)	Paillier	Fingerprint and iris (CASIA fingerprint and iris datasets)	-	Data: no information. Time: no information.
Barrero et al. [90] (2017)	Java-Paillier	Signature and fingerprint (BiosecurID DB)	Intel Core i7 with four 2.67 GHz cores.	Data: no information. Time: Comparison—0.5 ms.
Sperling et al. [91] (2022)	SEAL	Face and voice (CPLFW and Google Speech Commands)	-	Data: floating-point, 512 dimensions (voice) + 512 dimensions (face). Time: Score calculation per match—2.75 ms.

## Data Availability

Not applicable.
